# Evolution and textual quantitative analysis of China’s palliative care policies: a three-dimensional analytical framework approach

**DOI:** 10.3389/fpubh.2025.1624446

**Published:** 2025-09-02

**Authors:** Lele Wang, Shan Zhao, Furong Xing, Hanlu Zhang, Lei Qu, Dongli Li, Jingmin Ji, Jianya Ye

**Affiliations:** ^1^School of Nursing, Hebei University of Chinese Medicine, Shijiazhuang, China; ^2^Key Laboratory of Integrated Traditional Chinese and Western Medicine for Health and Care, Hebei University of Chinese Medicine, Shijiazhuang, China

**Keywords:** palliative care, policy tools, participants, time series, three-dimensional analysis

## Abstract

**Background:**

To summarize the evolution trend of China’s hospice policy, dissect the possible instrumental problems of the policy, and provide reference for the optimization and improvement of China’s hospice policy.

**Methods:**

Policy document analysis was used to encode and examine palliative care policy texts. The use of policy tools was assessed across three dimensions: policy types, participating entities, and temporal trends.

**Results:**

A total of 36 policy documents were analyzed. In the X dimension, 253 codes were identified, with environmental, supply, and demand policy tools comprising 57.71, 23.32, and 18.97%, respectively. In the Y dimension, 423 codes were identified, with government agencies, medical institutions, social organizations, and healthcare professionals comprising 73.05, 18.44, 4.96, and 3.55%, respectively. In the Z dimension, 253 codes were identified, corresponding to three stages: initial exploration (10.67%), in-depth exploration (14.62%), and pilot implementation (74.70%).

**Conclusion:**

Palliative care in China is gradually advancing. However, policy tools remain imbalanced, with a strong emphasis on environmental tools over demand-side tools, leading to internal structural inconsistencies. The allocation of policy tools among stakeholders is also uneven, characterized by a single-entity dominance and a government-driven supply model. It is recommended that policies be aligned with their developmental stages, the structure of policy tools be optimized, balanced utilization among stakeholders be ensured, and role differentiation and collaboration enhanced.

## 1 Introduction

Hospice care involves providing physical, psychological, spiritual, and humanistic support to patients with end-stage diseases or older adults nearing the end of life. Its primary goals are to manage pain and discomfort, enhance quality of life, and ensure patients pass away with dignity, comfort, and peace (1). With economic development, increased life expectancy, and a rising prevalence of chronic diseases, people are paying more attention to end-of-life quality. Consequently, the demand for hospice care in China is substantial, yet less than 7% of patients receive hospice services annually (2). The gap between the growing demand and the insufficient hospice resources highlights a critical imbalance in the service system. To address this issue, the government has implemented policies to expand hospice services, improve care models, enhance capacity, and bridge the supply–demand gap, ultimately improving end-of-life care quality. By 2023, China’s hospice care initiatives had made progress, with three batches of national pilot programs covering 185 cities and districts, gradually expanding from the provincial to the municipal level. However, challenges remain, including a limited number of service providers, a shortage of professionals, inconsistent service quality, and regional disparities in development (3). At this stage, a systematic analysis of China’s hospice policies is urgently needed to identify key focus areas, assess existing challenges, and propose more effective strategies. As a crucial tool for achieving governmental objectives, ensuring policy implementation, and enhancing policy effectiveness (4), the rational selection and scientific design of policy instruments play a decisive role in hospice care development. However, limited research has examined China’s hospice policies from the perspective of policy tools. This study aims to construct a three-dimensional policy analysis framework “policy tools - participating entities - time series” to analyze hospice policy texts quantitatively. It seeks to examine the use of policy tools at different stages, assess stakeholder distribution, and identify practical issues in current policies, ultimately providing insights for policy optimization. To optimize the hospice policy, we provide a reference (1) data and methods.

## 2 Materials and methods

### 2.1 Data sources and study selection criteria

This study systematically searched government websites, including those of the State Council and the National Health Commission, and policy databases such as the Peking University Legal Database and Lawstar. The core search terms “hospice care,” “end-of-life care,” “palliative care,” and “comfort care” were used to collect comprehensive national-level policy documents. The search period spanned documents published between September 1, 1994, and December 23, 2024. Policy texts were screened based on the following criteria, which involved reviewing their titles and content: (1) the policy document must originate from the national level, excluding those issued by local departments. (2) The document must be directly related to hospice care, excluding those containing only keywords without substantive measures. (3) Eligible policy types included opinions, plans, guidelines, and notices, whereas approvals, replies, work reports, public notices, and policy interpretations were excluded. Ultimately, 36 hospice care-related policy documents were identified (see Table 1).

**Table 1:** List of textual elements of our hospice policy, 1994–2024 (filtered).

Serial number	Policy document nomenclature	Issuance date	Issuing authority
1	Trial Standards for Medical Institutions (Trial Implementation)	1994.09	Former Ministry of Health
2	Notice on Distributing the “Catalog of Diagnosis and Treatment Subjects in Medical Institutions”	1994.09	Former Ministry of Health
3	Development Outline for China’s Nursing Career (2005–2010)	2005.07	Former Ministry of Health
4	Management Measures for Urban Community Health Service Institutions (Trial Implementation)	2006.08	Former Ministry of Health
5	Basic Standards for Nursing Homes (2011 Edition)	2011.03	Former Ministry of Health
6	Development Outline for China’s Nursing Career (2011–2015)	2011.12	Former Ministry of Health
7	State Council Opinions on Promoting the Development of Health Service Industry	2013.09	General Office of the State Council
8	Guiding Opinions on Promoting the Integration of Medical and Elderly Care Services	2015.11	General Office of the State Council
9	Healthy China 2030 Planning Outline	2016.10	General Office of the State Council
10	National Nursing Career Development Plan (2016–2020)	2016.11	Former National Health and Family Planning Commission
11	13th Five-Year Plan for Health and Healthcare	2017.01	General Office of the State Council
12	Basic Standards and Management Specifications for Palliative Care Centers (Trial Implementation)	2017.02	Former National Health and Family Planning Commission
13	Guidelines for Palliative Care Practice (Trial Implementation)	2017.02	Former National Health and Family Planning Commission
14	13th Five-Year Plan for Healthy Aging	2017.03	Former National Health and Family Planning Commission
15	State Council Opinions on Supporting Non-Governmental Entities in Providing Multi-Level and Diversified Medical Services	2017.05	General Office of the State Council
16	State Council Guidelines on Further Reforming the Payment Methods of Basic Medical Insurance	2017.06	General Office of the State Council
17	Notice on Launching the First Batch of Palliative Care Pilot Programs	2017.10	Former National Health and Family Planning Commission
18	Joint Notice on Promoting the Reform and Development of Nursing Service Industry	2018.07	National Health Commission et al.
19	State Council Notice on Key Tasks for Deepening Healthcare System Reform in the Second Half of 2018	2018.08	General Office of the State Council
20	Notice on Launching the Second Batch of Palliative Care Pilot Programs	2019.05	National Health Commission
21	Guiding Opinions on Establishing and Improving the Elderly Health Service System	2019.10	National Health Commission et al.
22	National Medium-to-Long Term Plan for Active Response to Population Aging	2019.11	General Office of the State Council
23	Notice on Strengthening Geriatric Nursing Services	2019.12	National Health Commission
24	Notice on Issuing Service Guidelines for Integrated Medical and Elderly Care Institutions (Trial Implementation)	2019.12	National Health Commission
25	Notice on Launching Pilot Programs for Geriatric Medical and Nursing Services	2020.11	National Health Commission
26	Notice on Issuing the 14th Five-Year Plan for National Aging Undertakings and Elderly Care Service System	2021.12	General Office of the State Council
27	Guiding Opinions on Further Promoting the Development of Integrated Medical and Elderly Care Services	2022.07	National Health Commission et al.
28	Notice on Issuing the 14th Five-Year Plan for National Health	2022.11	General Office of the State Council
29	Notice on Launching the Third Batch of Palliative Care Pilot Programs	2023.07	National Health Commission
30	Notice on Issuing Service Guidelines for Integrated Home and Community Medical and Elderly Care Services (Trial Implementation)	2023.11	National Health Commission et al.
31	Opinions on Developing Silver Economy to Improve Elderly Well-being	2024.01	General Office of the State Council
32	Notice on Issuing Reference Standards for the Construction of Key Central Township Hospitals	2024.07	National Health Commission et al.
33	Announcement on Publishing Two Recommended Health Industry Standards Including “Setting Standards for Geriatric Palliative Care Wards”	2024.08	National Health Commission
34	Guidelines for Establishing Comprehensive Diagnostic Medical Service Price Items (Trial Implementation)	2024.11	National Healthcare Security Administration
35	Notice on Enhancing Geriatric Medical Service Capabilities	2024.11	National Health Commission
36	Guiding Opinions on Promoting High-Quality Development of Integrated Medical and Elderly Care Services	2024.12	National Health Commission

### 2.2 Policy three-dimensional analysis framework

#### 2.2.1 Policy instruments (X dimension)

This study employs the policy analysis framework developed by Rothwell and Zegveld, which classifies policy instruments into three categories: supply-side, environment-side, and demand-side. This framework is widely recognized in academia for its clear definitions and practical applicability in classifying policy instruments (5). Building on this theory and considering the characteristics of hospice care, we have refined the definitions of the three policy instrument types to enhance the relevance of hospice care policy analysis (see Table 2). Specifically, supply-side policy instruments involve the government actively providing resources—such as human capital, financial support, and information technology—by leveraging its authority. Environment-side policy instruments primarily shape hospice care by fostering a supportive social environment through policies such as targeted planning and regulatory standards. Demand-side policy instruments leverage market mechanisms to drive the internal development of hospice care, incentivizing various entities to offer hospice services through policies such as pilot programs and medical insurance subsidies (6).

**Table 2:** Categories and specific meanings of hospice-related policy instruments.

Policy type	Instrument name	Description
Supply-side policy instruments	Infrastructure construction	Government-backed expansion of Hospice care facilities
	Talent cultivation	Implement comprehensive hospice care training programs to strengthen Hospice care delivery capabilities
	information support	Encourage agencies to integrate information resources, realize information sharing, and provide information and technical support for the development of hospice services
	Financial support	Specialized government funding for hospice institutions
Environmental policy instruments	Standard and regulation	Government develops hospice standards, guidelines to standardize hospice services
	supervision and evaluation	The government establishes a hospice oversight and assessment and quality evaluation system to ensure that hospice care is implemented efficiently and with high quality
	Goal programming	The government has made a general plan and objectives for the development of hospice care
	Policy Communication	Strengthening policy training and publicity guidance, organizing and implementing hospice pilot projects, summarizing pilot experiences in a timely manner, and promoting the high-quality development of hospice care
Demand-side policy instrument	Service Delivery System	The Government gradually promotes the formation of a hospice service system that covers the pilot areas, with multiple organizing bodies and various forms of services
	Pilot Demonstration Program	The Government has steadily expanded the hospice pilot, summarized and promoted the experience and model of hospice services, and formed a demonstration role
	International Exchange and Cooperation	Strengthen international cooperation and deeply participate in the research and development of international standards, norms and guidelines related to hospice care
	Health Insurance Support	The government improves the universal health insurance system to provide protection for the development of hospice services
	Government purchase	The Government stimulates the demand for hospice services among the various actors by purchasing services and allocating the corresponding funds to the organizations that provide hospice care

#### 2.2.2 Stakeholders (Y dimension)

Policy formulation and implementation depend on designated institutions and accountable personnel (7). Policy analysis identifies four key stakeholder groups essential for effective hospice care implementation: healthcare institutions, government agencies (subdivided by function into departments overseeing medical insurance, information technology, finance, regulation, justice, public health, and publicity), social organizations, and healthcare professionals. These stakeholders collectively form the Y dimension of our analytical framework.

#### 2.2.3 Time series (Z-dimension)

To examine the evolution and optimization of policy instruments in palliative care, this study categorizes the policy timeline into three phases based on key policy milestones: the initial stage (1994–2011), the exploratory stage (2012–2016), and the pilot-led development stage (2017–2024). Policy evolution began in 1994 when the “Regulations on the Management of Medical Institutions” incorporated hospice care departments into the “Catalog of Medical Diagnosis and Treatment Subjects,” officially recognizing hospice care as an independent medical discipline in China. A key milestone occurred in 2012 with the enactment of the “Law of the People’s Republic of China on the Protection of the Rights and Interests of the Elderly,” which required relevant departments to provide palliative care services and encouraged eligible regions to explore service models-marking the first legal recognition of palliative care. The current phase began in October 2017, when the central government launched the first national palliative care pilot program in five cities and districts, including Haidian District in Beijing, initiating the systematic nationwide implementation of palliative care services.

#### 2.2.4 Overall framework

**Figure 1:**
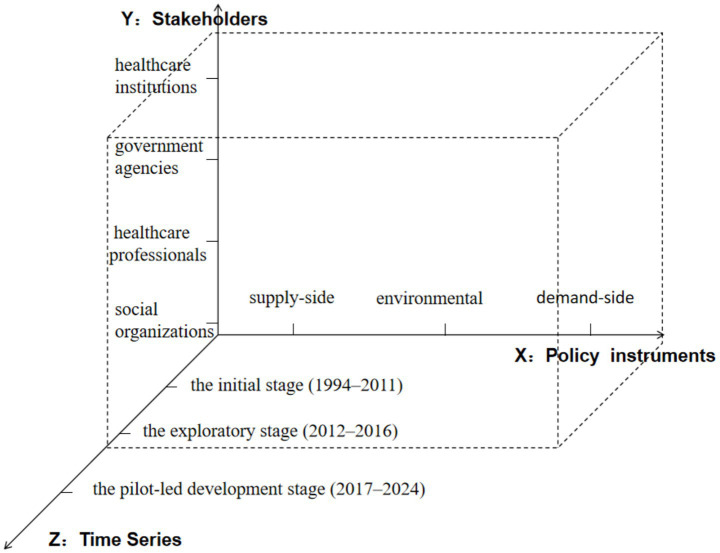
Three-dimensional analytical framework for hospice policy.

This study develops a three-dimensional analytical framework to examine China’s palliative care policies by deconstructing three core elements: policy instruments, stakeholders, and policy evolution stages. This framework serves as the foundation for policy text coding, facilitating a comprehensive analysis of palliative care policies (see Figure 1).

### 2.3 Data analysis methodology

This study examined 36 hospice and palliative care policies. Policy texts were imported into NVivo 14, using the smallest original sentence or paragraph as the unit of analysis. Each unit was coded and labeled with nodes based on predefined categories. The coding process followed the principles of consistency and indivisibility. If a content unit contained multiple policy tools or stakeholders, it was coded multiple times to ensure research accuracy and rigor. To reduce potential biases, two trained researchers conducted the coding under a supervisor’s guidance. After the initial coding, the results were cross-checked. Ambiguous or vaguely defined policy provisions were identified and recorded after team discussions. The coded data were categorized, organized, and analyzed using frequency statistics in Excel.

## 3 Results

### 3.1 Textual analysis findings

#### 3.1.1 Descriptive statistics of basic policy instruments along the X dimension

A total of 253 policy document coding units were identified for the X dimension, highlighting a significant imbalance in the coding distribution. Environmental policy instruments constituted the largest share, with 146 units (57.71%). This suggests that the government prioritizes fostering a supportive external environment to advance hospice and palliative care. Among the secondary indicators, target planning accounted for the highest share (24.51%), followed by standards and regulations (19.76%). This reflects the government’s efforts to define industry development directions through top-level design while ensuring service quality via standardized systems. Demand-side policy instruments were the least utilized. Among secondary indicators, service systems had the highest share (7.11%), whereas international exchange had the lowest (1.98%). Detailed statistical results are shown in Table 3. These findings indicate persistent imbalances and gaps in the use of policy instruments, highlighting the need for better integration and coordination.

**Table 3:** Distribution of basic policy tools.

Policy type	Total(n)	Proportion (%)	Instrument name	Number of policy codes (n)	Proportion (%)
Supply-side policy instruments	59	23.32	Infrastructure construction	10	3.95
			Talent cultivation	21	8.30
			information support	16	6.32
			Financial support	12	4.74
Environmental policy instruments	146	57.71	Standard and regulation	50	19.76
			supervision and evaluation	19	7.51
			Goal programming	62	24.51
			Policy Communication	15	5.93
Demand-side policy instrument	48	18.97	Service Delivery System	18	7.11
			International Exchange and Cooperation	5	1.98
			Pilot Demonstration Program	12	4.74
			Health Insurance Support	7	2.77
			Government purchase	6	2.37

#### 3.1.2 Statistical results of Y-dimensional participants

A total of 423 policy texts were coded based on the statistical results of the Y dimension. Policy texts related to government departments were the most prevalent, comprising 73.05%, followed by those involving medical institutions at 18.44%. Policy texts related to social organizations and medical staff were less frequent, representing 4.96 and 3.55%, respectively. This highlights a “strong government leadership, weak multi-sector coordination” pattern in the formulation and implementation of palliative care policies in China.

#### 3.1.3 Statistical results of policy development stages in dimension Z

A quantitative analysis examined the use of policy instruments across different stages of hospice and palliative care policy development. The distribution of policy instruments across the initial, exploratory, and pilot stages was 10.67, 14.62, and 74.70%, respectively. These findings indicate an increasing commitment from policymakers to advancing hospice and palliative care.

#### 3.1.4 Cross-analysis based on policy tools and participating subjects

A cross-analysis of policy tools and participating subjects was conducted to assess how each subject utilizes policy tools, providing insight into their roles in formulating and implementing hospice and palliative care policies. The overall distribution is presented in Table 4. Among environmental policy tools, those involving the health department are the most prevalent, whereas those involving social organizations, medical staff, and other departments are relatively scarce. In demand-and supply-oriented policies, the distribution of policy tools among subjects mirrors that of environmental policies, with those involving the health department being the most predominant. The allocation of policy tools among participating subjects is unbalanced, reflecting a predominantly top-down, government-led supply structure.

**Table 4:** Two-dimensional distribution of hospice policy tools (X-Y).

Policy type	Stakeholders										
	Healthcare professional	Healthcare institution	Social organization	Government agencies							
				Information technology	Medical Insurance	Health	Judicial	Advocacy	Supervision	Finance	Other
Supply-side											
Infrastructure construction	0	8	0	0	0	9	0	0	0	1	1
Talent cultivation	11	13	0	0	0	19	2	0	0	1	9
information support	0	1	1	16	0	5	0	0	0	0	0
Financial support	0	1	0	0	1	6	0	0	0	12	1
Environmental											
Standard and regulation	3	15	0	0	3	38	14	0	2	1	3
supervision and evaluation	0	2	0	0	0	9	1	0	18	0	0
Goal programming	1	25	19	0	3	44	2	0	0	3	9
Policy Communication	0	0	0	0	0	5	0	15	0	0	1
Demand-side											
Service Delivery System	0	10	0	0	0	18	0	0	0	0	0
International Exchange and Cooperation	0	0	0	0	0	5	0	0	0	0	2
Pilot Demonstration Program	0	3	0	0	2	10	0	0	0	0	0
Health Insurance Support	0	0	1	0	7	1	0	0	0	1	0
Government purchase	0	0	0	0	1	3	1	0	0	1	3

#### 3.1.5 Two-dimensional statistical results of policy tools and development stages

A bifurcation analysis was performed on two dimensions: policy tools and development stages, to examine how policy tools were utilized at each stage. The statistical results show a significant increase in the number of policy tools as development stages progress, reflecting policymakers’ growing emphasis on hospice and palliative care. This shift is most evident in the latest stages, where the distribution of tool types moved from a “single dominance” to “multiple collaborations,” indicating a trend toward more balanced proportions. Further analysis reveals that in the initial policy stages, the use of each tool was low, and tools like information support, medical insurance, and government procurement were not yet included, indicating an incomplete policy framework. During the development stages, both the diversity and frequency of policy tools improved, though structural imbalances in their use remained (Table 5).

**Table 5:** Two-dimensional distribution of hospice policy tools (X-Z).

Policy type	Time series					
	The initial stage (1994–2011)		The exploratory stage (2012–2016)		The pilot-led development stage (2017–2024)	
	Number of policy codes (n)	Proportion (%)	Number of policy codes (n)	Proportion (%)	Number of policy codes (n)	Proportion (%)
Supply-side	0	0.00	2	5.41	7	3.91
Infrastructure construction	2	7.41	4	10.81	15	7.94
Talent cultivation	0	0.00	4	10.81	12	6.70
information support	0	0.00	1	2.70	11	6.15
Financial support						
Environmental	6	22.22	3	8.11	41	21.69
Standard and regulation	2	7.41	4	10.81	13	7.26
Supervision and evaluation	14	51.85	12	32.43	36	20.11
Goal programming	0	0.00	1	2.70	14	7.82
Policy communication						
Demand-side	1	3.70	2	5.41	15	8.38
Service delivery system	2	7.41	1	2.70	2	1.12
International exchange and cooperation	0	0.00	1	2.70	11	6.15
Pilot Demonstration Program	0	0.00	1	2.70	6	3.35
Health Insurance Support	0	0.00	1	2.70	5	2.79
Total	27	100.00	37	100.00	189	100.00

## 4 Discussion

### 4.1 Overall status of hospice and palliative care policies

In response to the rapidly aging population and the growing demand for hospice and palliative care, the government has implemented a series of policies in this field. This study analyzes key issues in these policies from the perspective of policy instruments. Initially, the overall structure and internal components of policy instruments are imbalanced. There is an excessive reliance on environmental policy instruments, whereas demand-side and supply-side instruments remain underutilized. Secondly, the distribution of policy instruments among stakeholders shows significant disparities, primarily following a “government-led, medical institution-executed” linear implementation model. Effective multi-stakeholder collaboration mechanisms are still lacking. Furthermore, as hospice and palliative care continue to develop, policy instruments have undergone adjustments and optimizations over time. However, the imbalance in policy instrument utilization remains largely unchanged.

### 4.2 Imbalanced structure of policy instruments

Overall, the current government frequently employs environmental policy instruments, with some policy areas showing signs of oversaturation. Conversely, supply-side policy instruments, aimed at promoting development, and demand-side instruments, intended to stimulate demand, remain underutilized, resulting in an uneven policy distribution. Among environmental policies, target planning is the most widely used, suggesting that the government prioritizes guiding the direction and focus of hospice and palliative care through policy measures. However, most target plans remain vague and lack detailed development strategies and specialized policy content. Secondly, standards and regulations ensure the quality of hospice and palliative care by establishing a unified service framework. However, excessive reliance on standards and regulations reduces policy flexibility, weakening implementation effectiveness. In contrast, demand-side policy instruments are underdeveloped, failing to complement supply-side instruments effectively (8). Measures like medical insurance payments reduce patients’ financial burden, while government procurement strengthens service supply. Enhancing cooperation and exchanges facilitates learning from advanced international practices and improves hospice and palliative care services. However, the underutilization of instruments like government procurement and international exchanges diminishes their role in driving demand. Although supply-side policy instruments are relatively balanced internally, their overall quantity is limited, suggesting insufficient government support for hospice and palliative care infrastructure. This hampers the optimal allocation of resources (9).

### 4.3 The distribution of policy instruments among participating entities exhibits an imbalance

Disparities in policy instrument allocation arise from the diverse roles and functions of participating entities. Medical personnel are the primary providers of hospice and palliative care, while social organizations supplement medical resources, both playing essential roles in service delivery (10). However, policy instruments targeting these two entities are limited, suggesting inadequate attention to their role in policy formulation. This oversight undervalues their contributions to hospice and palliative care. Over time, this may reduce their motivation and hinder policy implementation. In contrast, healthcare institutions and the government possess the most policy instruments. These policies mainly emphasize environmental instruments, including admission standards for hospice centers, service management, operational guidelines, and standardized care practices to enhance institutional development. This reflects the current state of hospice and palliative care in China, which remains under development and faces significant challenges (11). Thus, without effective coordination of responsibilities, implementing numerous policies may fail to accelerate hospice and palliative care development.

## 5 Conclusion

### 5.1 Enhance the balance in the utilization of policy instruments and optimize the internal structure of policies

It is essential to balance the distribution of policy instruments. For environmental policy instruments, breaking path dependence and reducing overuse is essential, alongside addressing internal structural imbalances. Quality assessment and regulatory mechanisms should be established and improved, with regular inspections of designated hospice and palliative care institutions. Additionally, enhances policy advocacy by disseminating hospice and palliative care knowledge through multiple channels to improve public acceptance (12). Furthermore, enhances supply-side policy instruments to improve hospice and palliative care resource allocation. Policies should prioritize the training of hospice and palliative care professionals, offering support in staffing, employment, continuing education, and salaries to sustain workforce motivation (13). Adequate healthcare resource allocation and flow require prioritizing financial investment and infrastructure development. Additionally, information technology should be integrated into palliative care through a dedicated information system, facilitating digital management of patient records, treatment histories, and medical orders. Finally, strengthening demand-oriented policy tools is essential, leveraging pilot programs and summarizing experiences to facilitate gradual expansion. At the same time, reforms in the palliative care insurance payment system should be promoted. The scope of payment should be expanded, incorporating international experiences to gradually include innovative service models such as home care, outpatient treatment, and telemedicine in the insurance framework. The payment system should be optimized, primarily utilizing per diem payments with a diversified approach (14).

### 5.2 Collaboration among stakeholders should be strengthened, and multi-party participation should be encouraged

Effectively implementing hospice and palliative care policies requires a collaborative mechanism involving multiple stakeholders. Policymakers should consider the characteristics of each stakeholder and guide government departments, medical institutions, and social organizations to leverage their strengths in hospice and palliative care. First, administrative barriers and institutional constraints between departments should be removed to facilitate multi-departmental collaboration and ensure effective policy implementation. Second, a collaborative mechanism for multi-level medical institutions should be established, leveraging the leadership of large tertiary hospitals under national policy guidance. A long-term collaborative mechanism should be established via medical consortiums, linking secondary and tertiary hospitals with primary healthcare centers to integrate and distribute high-quality medical resources efficiently. Thirdly, a diversified collaborative system for hospice and palliative care should be developed. This involves establishing cross-industry collaboration platforms, encouraging structured social capital investment, and engaging non-profit organizations, such as charities, in hospice and palliative care development through special funds and targeted donations. This approach fosters a model driven by government guidance, market operations, and social participation.

### 5.3 Addressing the phase-specific characteristics of policy development: timely adjustment and innovation of policy instruments

Adapting to societal changes, medical advancements, and policy shifts requires timely adjustments and innovations in policy instruments to sustain hospice and palliative care development. Future policy instrument design and implementation require a comprehensive approach that integrates scientific forecasting, strategic planning, and deliberate policy tool design to enhance adaptability and sustainability. A systematic approach to policy instrument innovation is essential to mitigate the limitations of over-reliance on single instruments or imbalanced utilization. This requires a holistic approach that considers hospice and palliative care policy objectives, implementation pathways, and resource allocation to ensure alignment with relevant social security policies. The government must systematically integrate resources across departments and levels, fostering synergy among policy instruments to enhance overall effectiveness.
